# Retrospective Analysis of the Clinical Significance of Positive Blood Cultures in the Emergency Department: A Single-Center Study

**DOI:** 10.1093/ofid/ofaf352

**Published:** 2025-06-17

**Authors:** Farha Ahmed Karlath, Mehboob Ahmed Rehan, Annie Geiger, Michael J Mitchell, Sami Arnaout, Thomas C Greenough, Richard T Ellison

**Affiliations:** Department of Medicine, Division of Infectious Diseases & Immunology, UMass Chan Medical School, Worcester, Massachusetts, USA; Department of Medicine, Division of Infectious Diseases & Immunology, United Regional Healthcare System, Wichita Falls, Texas, USA; UMass Chan Medical School, Worcester, Massachusetts, USA; Department of Pathology, UMass Chan Medical School, Worcester, Massachusetts, USA; Department of Medicine, Division of Infectious Diseases & Immunology, UMass Chan Medical School, Worcester, Massachusetts, USA; Department of Medicine, Division of Infectious Diseases & Immunology, UMass Chan Medical School, Worcester, Massachusetts, USA; Department of Medicine, Division of Infectious Diseases & Immunology, UMass Chan Medical School, Worcester, Massachusetts, USA; Department of Microbiology and Physiological Systems, UMass Chan Medical School, Worcester, Massachusetts, USA

**Keywords:** blood culture, CoNS, contaminant

## Abstract

**Background:**

There have been major advances in blood culture technology in the last decade, with both faster and more sensitive pathogen detection as well as more precise species identification. We have reassessed the results of positive blood cultures in this new clinical microbiology era with a focus on contaminant identification.

**Methods:**

A retrospective study was conducted including all patients with a blood culture collected in 2 UMass Memorial Health emergency departments from September 2019 through April 2020. Contaminants were identified based on standard clinical microbiology laboratory criteria and independent retrospective review by 3 infectious disease (ID) physicians and an ID fellow.

**Results:**

Of 5673 blood samples obtained, 5661 were analyzed after 12 were deemed inconclusive by the ID physician review. Blood culture contaminants accounted for 22.5% of the positive blood cultures. *Staphylococcus epidermidis* was the most frequent contaminant (33.4%), while *Escherichia coli* was the most frequent pathogen (21%) causing true bacteremia. Coagulase-negative staphylococci remain the most frequent cause of blood culture contamination with *S epidermidis* being the most common. The *Staphylococcus* species *S auricularis, S caprae, S lentus, S pseudointermedius, S saccharolyticus,* and *S warneri* were all determined to be contaminants in 100% of cases.

**Conclusions:**

The improvements in clinical laboratory technology enable better discrimination of the relative pathogenicity of differing coagulase-negative staphylococci species.

Blood culture contamination remains a significant clinical issue, particularly in emergency department (ED) settings. Recent advancements in blood culture diversion devices have shown promise in reducing contamination rates, thereby improving diagnostic accuracy and patient outcomes. Arenas [1] demonstrated that the use of 2 different specimen diversion devices significantly reduced blood culture contamination rates compared with traditional methods, highlighting the effectiveness of these devices in clinical practice. Similarly, Tompkins et al [2] reported that the implementation of an initial specimen diversion device (ISDD) substantially reduced blood culture contamination and false-positive central line–associated bloodstream infections, further supporting the utility of these devices in both inpatient and ED settings.

The introduction of advanced blood culture systems and rapid diagnostic methods has revolutionized pathogen identification and antimicrobial susceptibility testing. Lamy et al [3] reviewed the progress in blood culture diagnostics, emphasizing the importance of optimizing preanalytical parameters and incorporating rapid methods such as matrix-assisted laser desorption/ionization time-of-flight (MALDI-TOF) mass spectrometry (MS) for timely and accurate pathogen identification. These advancements underscore the need for continuous improvement in blood culture practices to enhance patient care and diagnostic stewardship.

Several prior assessments of the clinical significance of positive blood cultures have noted changes over time likely related to both changes in laboratory technology and major advances in medical care in relation to the introduction of new vaccines, increasing antimicrobial resistance, and an increasing immunocompromised population [4–7]. In light of these developments, our study aims to evaluate the impact of blood culture diversion devices on contamination rates and diagnostic accuracy and to analyze the significance of positive blood cultures in the era of advanced microbiological technologies.

## METHODS

This is a single-center retrospective study of patients with a blood culture collected in the UMass Memorial Medical Center University and Memorial Campus EDs from September 2019 through April 2020 during a prospective controlled trial of a passive blood diversion device to prevent blood culture contamination [8]. All blood culture samples collected from the 2 EDs during the study period were included in the study. Pediatric blood culture samples were excluded due to the presence of dedicated pediatric staff at only one of the campuses. Samples were also excluded from analysis if ID physician review could not determine conclusively whether results represented true bacteremia or contaminants. The University Campus, a level 1 trauma center, also includes a stroke center, interventional cardiology/cardiac surgery centers, a pediatric intensive care unit, and transplant services. In contrast, the Memorial Campus offers general medical/surgical services, elective orthopedic surgeries, and obstetrical/gynecological services.

Blood culture samples were analyzed using the (BacT/ALERT VIRTUO; bioMerieux) automated detection system, with identification to the species level of microbial isolates performed using Clinical and Laboratory Standards Institute standard techniques, including MALDI-TOF MS (Vitek MS Maldi-TOF; bioMerieux). An episode of bacteremia was defined as the first positive blood culture or a new positive blood culture, which must have occurred ≥2 days after any previous positive blood culture [4].

Positive blood cultures considered as likely contaminants were determined both according to standard laboratory definitions [9, p 110] and by record review by 3 independent infectious diseases (ID) physicians and an ID fellow considering the patient's reported clinical history, physical findings, laboratory and imaging findings, number of positive blood cultures of the total number performed, time to positivity of the blood cultures, clinical course, and response to therapy. An interrelated reliability score was calculated. In practice, a positive blood culture was more likely to be considered a contaminant if it consisted of ≥1 of the typical skin bacteria growing in 1 of 4 culture bottles, in the absence of fever and a clear source, occasionally accompanied by chart documentation of difficult blood sampling. Standard laboratory practice defined blood culture contamination as any culture for which low-virulence organisms typical of normal mucocutaneous flora are isolated from a single culture of all blood culture sets obtained from a single patient on the same day (eg, coagulase-negative staphylococci [CoNS], α-hemolytic streptococci, and *Micrococcus, Cutibacterium* [*Propionibacterium*]*, Corynebacterium*, and *Bacillus* species) [9, p 110].

## RESULTS

During the study period, 5637 blood culture bottles were collected, 624 with microbial growth. Twelve samples with bacterial growth were excluded from analysis as ID physician review could not determine conclusively whether results represented true bacteremia or contaminants. Among 159 samples that met the standard laboratory criteria to be considered blood culture contaminants, ID physician review identified 27 (17%) that were clinically considered to represent a true infection; among 453 samples identified as a true pathogen by laboratory criteria, only 6 (1%) were deemed by ID physician review to be a blood culture contaminant. The overall blood culture contaminant rate across both EDs combined was 2.4%. There was an interrelated reliability score of 95% using the percentage agreement method.

Approximately 56% of adult patients with true bloodstream infections were male, and 16.5% were immunocompromised. Patients were classified as immunocompromised based on criteria outlined by the Centers for Disease Control and Prevention. These include individuals undergoing active treatment for solid tumor or hematologic cancer, those who have received a solid organ transplant and are taking immunosuppressive therapy, and those receiving chimeric antigen receptor T-cell therapy or hematopoietic stem cell transplants (within 2 years of transplantation or while receiving immunosuppressive therapy). Individuals with moderate or severe primary immunodeficiency or advanced or untreated human immunodeficiency virus (HIV) infection and those receiving high-dose corticosteroids, alkylating agents, antimetabolites, or other immunosuppressive therapies were also considered immunocompromised. Among patients with true bacteremia, diabetes mellitus was noted as the most common comorbid condition, accounting for up to 39.4%, followed by coronary artery disease (24.5%) (Table 1).

**Table 1.** Characteristics of Patients With Positive Blood Cultures

Characteristics	Patients, No. (%)
Sex	
Male	364 (56.3)
Female	282 (43.7)
Immunosuppression^a^	
Yes	108 (16.5)
No	548 (83.5)
Comorbid conditions	
Diabetes mellitus	259 (39.4)
COPD	94 (14.3)
Coronary artery disease	161 (24.5)
Rheumatoid arthritis	32 (4.9)
SLE	8 (1.2)
Multiple sclerosis	22 (3.4)
Dialysis	39 (5.9)
Presence of foreign body (mechanical valve/prosthetic joint)	85 (13)
Long-term Foley/suprapubic catheter	80 (12.2)
Central line/portacath	44 (6.7)

Abbreviations: COPD, chronic obstructive pulmonary disease; SLE, systemic lupus erythematosus.

^a^Patients were classified as immunocompromised based on criteria outlined by the Centers for Disease Control and Prevention. These include individuals undergoing active treatment for solid tumors or hematologic cancers, those who have received a solid organ transplant and are receiving immunosuppressive therapy, and those receiving chimeric antigen receptor T-cell therapy or hematopoietic stem cell transplants (within 2 years of transplantation or while on immunosuppressive therapy). Individuals with moderate or severe primary immunodeficiency, advanced or untreated human immunodeficiency virus infection, or those receiving high-dose corticosteroids, alkylating agents, antimetabolites, or other immunosuppressive therapies were also considered immunocompromised.

The most frequently isolated microorganisms causing episodes of true bacteremia were *Escherichia coli* (105 episodes), *Staphylococcus aureus* (99 episodes, including 30 episodes of methicillin-resistant and 69 of methicillin-susceptible *S aureus*), *Klebsiella pneumoniae* (36 episodes), and *Enterococcus* species (32 episodes, including 24 of *Enterococcus faecalis*, 7 of *Enterococcus faecium*, and 1 of *Enterococcus avium*). Most positive blood culture episodes due to *E coli* and other Enterobacteriaceae, *S aureus, Streptococcus pneumoniae,* and *Streptococcus pyogenes* were confirmed as true bloodstream infections. Most viridans streptococci were also determined to cause true bloodstream infections. Notably, *Staphylococcus epidermidis* grew from 111 positive blood cultures, with only 10 confirmed as true bloodstream infections.

**Figure 1.**
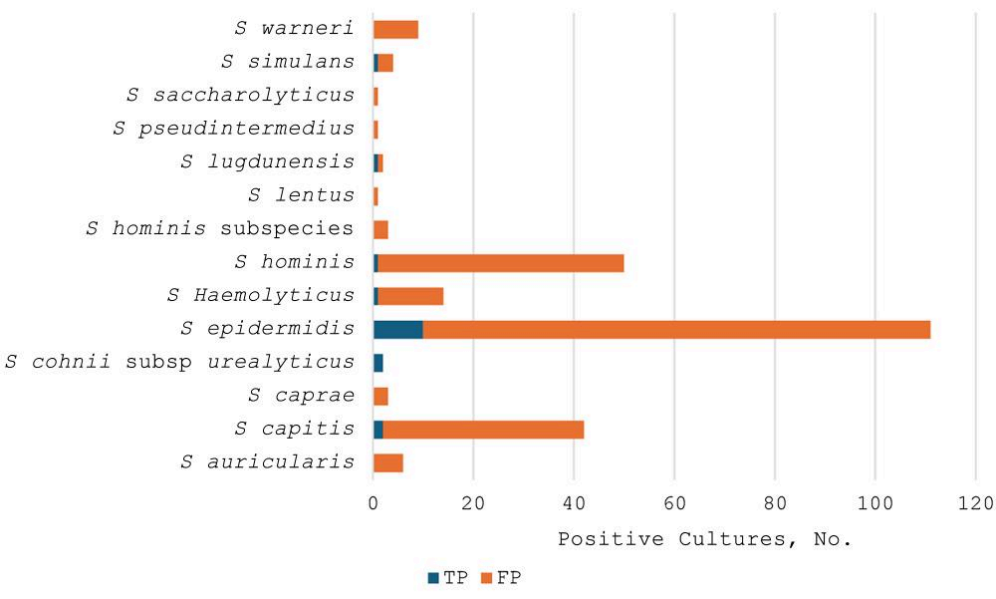
Comparison of various species of coagulase-negative *Staphylococcus* with positive (false-positive [FP] or true-positive [TP]) blood cultures.

Similarly, most other species of CoNS, including *Staphylococcus auricularis*, *Staphylococcus capitis*, *Staphylococcus caprae*, *Staphylococcus haemolyticus*, *Staphylococcus hominis*, *S hominis* subsp *hominis*, *Staphylococcus lentus*, *Staphylococcus lugdunensis*, *Staphylococcus pseudintermedius*, *Staphylococcus saccharolyticus*, *Staphylococcus simulans*, and *Staphylococcus warneri,* were considered contaminants, except for *Staphylococcus cohnii* subspecies *urealyticus*, which was found in 2 positive blood cultures, both confirmed as true bloodstream infections. Among CoNS, *S auricularis*, *S caprae*, *S lentus*, *S pseudintermedius*, *S saccharolyticus*, and *S warneri* were all determined to be contaminants in 100% of cases. The majority of blood cultures growing *Corynebacterium* and *Bacillus* species were also considered contaminated (Table 2 and Figure 1).

**Table 2.** Microorganisms Isolated From Positive Blood Cultures

Microorganism	Isolates, No.			Source of TP Bacteremia, No. of Isolates										
	Total	TP	FP	GI	GU	Skin	Skin (SS)	Skin (IVDU)	Pulm	CV	Neuro	MSK	VC	ND
*Abiotrophia defectiva*	1	1	0	…	…	…	…	…	…	1	…	…	…	…
*Acinetobacter calcoaceticus-baumannii* complex	1	1	0	…	…	…	…	…	…	…	…	…	1	…
*Acinetobacter lwoffi*	3	1	2	…	…	1	…	…	…	…	…	…	…	…
*Actinomyces naeslundii*	2	1	1	…	…	1	…	…	…	…	…	…	…	…
*Actinomyces odontolyticus*	1	1	0	1	…	…	…	…	…	…	…	…	…	…
*Actinotignum schaalii*	1	1	0	…	1	…	…	…	…	…	…	…	…	…
*Aerococcus viridans*	3	2	1	…	…	1	…	…	…	…	1	…	…	…
*Anaerococcus prevotii*	2	0	2	…	…	…	…	…	…	…	…	…	…	…
*Bacillus cereus*	1	1	0	…	…	…	…	…	…	…	…	…	…	1
*Bacillus* sp	6	1	5	…	…	…	…	…	…	…	…	…	…	1
*Bacteroides* species														
* B caccae*	1	1	0	1	…	…	…	…	…	…	…	…	…	…
* B fragilis*	3	3	0	3	…	…	…	…	…	…	…	…	…	…
* B ovatus*	1	1	0	1	…	…	…	…	…	…	…	…	…	…
* B thetaiotaomicron*	2	2	0	1	…	1	…	…	…	…	…	…	…	…
* B uniformis*	1	1	0	…	1	…	…	…	…	…	…	…	…	…
* B vulgatus*	2	2	0	2	…	…	…	…	…	…	…	…	…	…
*Bifidobacterium* sp	1	1	0	1	…	…	…	…	…	…	…	…	…	…
*Burkholderia cepacia* complex	1	1	0	…	…	…	…	…	…	…	…	…	1	…
*Citrobacter freundii*	3	3	0	…	3	…	…	…	…	…	…	…	…	…
*Citrobacter koseri*	1	1	0	…	1	…	…	…	…	…	…	…	…	…
*Clostridium* species														
* C clostridioforme*	1	1	0	…	…	…	…	…	…	…	…	1	…	…
* C perfringens*	2	2	0	2	…	…	…	…	…	…	…	…	…	…
* C ramosum*	2	1	1	1	…	…	…	…	…	…	…	…	…	…
* C septicum*	2	2	0	2	…	…	…	…	…	…	…	…	…	…
* C tertium*	1	1	0	1	…	…	…	…	…	…	…	…	…	…
*Corynebacterium* species														
* C amycolatum*	1	1	0	…	…	…	…	…	…	…	…	…	…	…
* C minutissimum*	1	0	1	…	…	…	…	…	…	…	…	…	…	…
Other species	16	3	13	…	…	1	…	…	…	1	…	…	…	1
* C striatum*	1	0	1	…	…	…	…	…	…	…	…	…	…	…
*Enterobacter cloacae*	8	8	0	2	3	1	…	…	1	…	…	1	…	…
*Enterobacter gergoviae*	1	1	0	…	…	…	…	…	…	…	…	…	…	1
*Enterococcus* species														
* E avium*	1	1	0	…	1	…	…	…	…	…	…	…	…	…
* E casseliflavus*	1	0	1	…	…	…	…	…	…	…	…	…	…	…
* E faecalis*	24	24	0	7	10	…	…	…	…	2	…	1	2	2
* E faecium*	7	7	0	5	…	…	1	…	…	1	…	…	…	…
*Escherichia coli*	105	105	0	30	73	…	…	…	1	…	…	…	1	…
*Eubacterium* sp	1	1	0	1	…	…	…	…	…	…	…	…	…	…
*Finegoldia magna*	3	3	0	…	2	…	…	…	…	…	…	…	…	1
*Fusobacterium nucleatum*	2	1	1	1	…	…	…	…	…	…	…	…	…	…
Other *Fusobacterium* species	1	1	0	…	1	…	…	…	…	…	…	…	…	…
*Gemella morbillorum*	1	1	0	1	…	…	…	…	…	…	…	…	…	…
*Globicatella sanguinis*	3	2	1	…	…	…	…	…	…	…	1	…	…	1
*Granulicatella adiacens*	2	2	0	…	…	…	…	…	1	…	1	…	…	…
*Granulicatella elegens*	0	0	0	…	…	…	…	…	…	…	…	…	…	…
GAS	5	5	0	1	…	3	…	…	…	…	…	…	…	1
GBS	8	8	0	…	…	4	…	…	…	1	…	1	…	2
GCS	1	1	0	…	…	1	…	…	…	…	…	…	…	…
GGS	3	3	0	1	…	1	…	…	…	…	…	…	…	1
*Haemophilus influenzae*	3	3	0	…	…	…	…	…	1	…	…	…	…	2
*Haemophilus parainfluenzae*	1	1	0	…	…	…	…	…	…	…	…	…	…	1
*Klebsiella oxytoca*	5	5	0	2	1	…	…	…	2	…	…	…	…	…
*Klebsiella pneumoniae*	36	36	0	15	19	…	…	…	…	…	…	…	2	…
MRSA	30	30	0	1	…	4	1	7	7	4	…	3	3	4
MSSA	69	69	0	2	1	14	2	6	8	5	…	15	9	7
*Microbacterium hydrothermale/testaceum/chocolatum*	1	0	1	…	…	…	…	…	…	…	…	…	…	…
*Micrococcus luteus*	9	0	9	…	…	…	…	…	…	…	…	…	…	…
*Micrococcus* sp	1	0	1	…	…	…	…	…	…	…	…	…	…	…
*Moraxella* sp	2	2	0	…	1	…	…	…	1	…	…	…	…	…
*Morganella morganii*	2	2	0	…	2	…	…	…	…	…	…	…	…	…
*Pantoea agglomerans*	1	1	0	…	…	…	…	…	…	…	…	…	1	…
*Pasteurella multocida*	2	2	0	…	1	…	…	…	…	…	…	…	…	1
*Peptoniphilus asaccharolyticus*	1	1	0	…	1	…	…	…	…	…	…	…	…	…
*Peptostreptococcus micros*	1	0	1	…	…	…	…	…	…	…	…	…	…	…
*Peptostreptococcus* species	5	4	1	4	…	…	…	…	…	…	…	…	…	…
*Propionibacterium acnes*	3	1	2	…	…	…	…	…	…	…	…	…	…	1
*Proteus mirabilis*	12	12	0	…	10	1	…	…	…	…	…	…	…	1
*Providencia rettgeri*	1	1	0	…	…	…	…	…	…	…	…	1	…	…
*Providencia stuartii*	2	2	0	…	2	…	…	…	…	…	…	…	…	…
*Pseudomonas aeruginosa*	9	9	0	3	5	…	…	…	…	…	…	…	1	…
*Rothia mucilaginosa*	1	1	0	…	…	…	…	1	…	…	…	…	…	…
*Serratia marcescens*	13	13	0	2	2	…	1	1	2	…	…	1	1	3
*Staphylococcus species*														
* S auricularis*	6	0	6	…	…	…	…	…	…	…	…	…	…	…
* S capitis*	42	2	40	…	…	…	…	…	…	1	…	…	1	…
* S caprae*	3	0	3	…	…	…	…	…	…	…	…	…	…	…
* S cohnii* subsp *urealyticus*	2	2	0	…	…	1	…	…	…	1	…	…	…	…
* S epidermidis*	111	10	101	1	2	…	…	…	…	5	…	…	1	1
* S haemolyticus*	14	1	13	…	…	…	…	…	…	…	…	…	1	…
* S hominis*	50	1	49	1	…	…	…	…	…	…	…	1	…	…
* S hominis* subsp *hominis*	3	0	3	…	…	…	…	…	…	…	…	…	…	…
* S lentus*	1	0	1	…	…	…	…	…	…	…	…	…	…	…
* S lugdunensis*	2	1	1	…	…	1	…	…	…	…	…	…	…	…
* S pseudintermedius*	1	0	1	…	…	…	…	…	…	…	…	…	…	…
* S saccharolyticus*	1	0	1	…	…	…	…	…	…	…	…	…	…	…
* S simulans*	4	1	3	…	…	1	…	…	…	…	…	…	…	…
* S warneri*	9	0	9	…	…	…	…	…	…	…	…	…	…	…
*Streptococcus* species														
* S agalactiae*	14	14	0	2	2	1	1	…	2	2	…	…	1	3
* S anginosus*	4	4	0	2	…	…	…	…	1	…	…	1	…	…
* S constellatus*	3	3	0	1	2	…	…	…	…	…	…	…	…	…
* S crista*	1	1	0	…	…	…	…	…	…	…	…	…	…	…
* S gallolyticus* subsp *gallolyticus*	1	1	0	…	…	…	…	…	…	1	…	…	…	1
* S gallolyticus* subsp *pasteurianus*	1	1	0	1	…	…	…	…	…	…	…	…	…	…
* S gordonii*	1	1	0	1	…	…	…	…	…	…	…	…	…	…
* S intermedius*	1	0	1	…	…	…	…	…	…	…	…	…	…	…
* S mitis* group	18	15	3	8	…	1	…	1	…	3	…	1	1	…
* S parasanguinis*	4	3	1	2	…	…	…	…	…	…	…	…	1	…
* S pneumoniae*	9	9	0	…	…	1	…	…	8	…	…	…	…	…
* S pyogenes*	5	5	0	…	…	2	1	…	…	…	…	2	…	…
* S salivarius*	4	3	1	2	…	…	…	…	…	…	…	…	1	…
* S sanguis*	2	2	0	…	…	…	…	…	…	1	…	1	…	…
* S thermophilus*	1	0	1	…	…	…	…	…	…	…	…	…	…	…
Viridans group^a^	3	2	1	…	…	2	…	…	…	…	…	…	…	…
*Turicella otitidis*	1	0	1	…	…	…	…	…	…	…	…	…	…	…
*Veillonella* species	3	2	1	1	…	1	…	…	…	…	…	…	…	…
Not speciated														
GN bacilli	4	3	1	…	3	…	…	…	…	…	…	…	…	…
GP bacilli	7	1	6	…	…	…	…	…	1	…	…	…	…	…
GP cocci in clusters	6	1	5	…	…	…	…	…	…	1	…	…	…	…
GP cocci	1	1	0	…	…	…	…	…	1	…	…	…	…	…
GV bacilli	1	0	1	…	…	…	…	…	…	…	…	…	…	…
Anaerobic GP bacilli	1	1	0	1	…	…	…	…	…	…	…	…	…	…
Anaerobic GP cocci	2	1	1	…	…	…	…	…	…	…	…	1	…	…

Abbreviations: CV, cardiovascular; FP, false-positive; GAS, GBS, GCS, and GGS, group A, group B, group C, and group G *Streptococcus*; GI, gastrointestinal; GN, gram-negative; GP, gram-positive; GU, genitourinary; GV, gram-variable; IVDU, intravenous drug use; MSK, musculoskeletal; MRSA and MSSA methicillin-resistant and methicillin-susceptible *Staphylococcus aureus*; ND, could not be determined; Neuro, neurologic; Pulm, pulmonary; SS, surgical site; TPd, true-positive; VC, venous catheter.

^a^Reported as species group.

The patients with true-positive CoNS bacteremia were predominantly male (63.6%; 36.4% were female). A small percentage (6.3%) were immunosuppressed, and 3.1% had received a liver transplant, while none had received kidney, heart, lung, or pancreas transplants. Active cancer was present in 9.4%, and half of the patients had diabetes mellitus. Chronic obstructive pulmonary disease affected 6.3%, and 40.6% had coronary artery disease. Rheumatoid arthritis and multiple sclerosis were each present in 6.3% of patients. Dialysis was required by 18.8%, and 28.1% had a foreign body. Recent surgery was reported in 15.6%, 12.5% had a long-term Foley or suprapubic catheter, and 6.3% had an intravascular catheter (Table 3).

**Table 3.** Characteristics of Patients with True-Positive Coagulase-Negative Staphylococcal Bacteremia

Characteristics	Patients With True-Positive CoNS Bacteremia, %
Sex	
Male	63.6
Female	36.4
Immunosuppressed	6.3
Solid organ transplant	3.1
Liver	
Kidney	0
Heart	0
Lung	0
Pancreas	0
Active cancer	9.4
Diabetes mellitus	50
COPD	6.3
Coronary artery disease	40.6
Rheumatoid arthritis	6.3
SLE	0
Multiple sclerosis	6.3
Dialysis	18.8
Presence of foreign body	28.1
Recent surgery	15.6
Long-term Foley catheter/suprapubic catheter	12.5
Intravascular catheter	6.3

Abbreviations: CoNS, coagulase-negative staphylococci; COPD, chronic obstructive pulmonary disease; SLE, systemic lupus erythematosus.

The most frequently identifiable sources of true bacteremia were genitourinary (150 episodes), followed by gastrointestinal (117 episodes) and skin (68 episodes). Among skin infections as the source of bacteremia, 45 were due to primary skin infection, 7 to surgical site infection, and 16 to intravenous drug use. The source of infection could not be determined in 37 episodes. *E coli* was the pathogen most frequently isolated from bacteremia caused by genitourinary and gastrointestinal infections. After *E coli, K pneumoniae* and *Proteus mirabilis* most often caused bacteremia arising from genitourinary infections, and *K pneumoniae* and *Enterococcus* species most often caused those due to gastrointestinal infections. Gram-positive pathogens, including staphylococcal and streptococcal species, caused most of the skin infections leading to bacteremia (Table 2).

Bacterial growth was noted with Gram stain in 22 study samples, but species could not be identified, even with MALDI-TOF. The bacteria were deemed to be contaminants in 14 samples and true pathogens in 8, with the genitourinary tract the most frequent source (Table 2).

## DISCUSSION

Blood culture contamination remains a significant clinical challenge, leading to unnecessary interventions and increased healthcare costs. Despite the implementation of various strategies to reduce blood culture contamination, achieving the recommended target contamination rate remains challenging for many institutions. The Infectious Diseases Society of America and the American Society for Microbiology recommend meticulous skin preparation before venipuncture, with chlorhexidine gluconate and iodine tincture being superior to povidone-iodine [10]. Recent advancements, such as the use of ISDDs, have shown promise in reducing contamination rates. Our study evaluated the clinical significance of blood cultures analyzed using MALDI-TOF technology, with a focus on CoNS species. The results of the prospective controlled trial associated with this study affirm that use of the blood diversion device decreased blood culture contamination rates in busy EDs in both the intention-to-use and actual-use analyses.

Previous studies [4–7] have examined the clinical significance of blood culture growth, but the technology used to differentiate microbes has markedly evolved since these previous studies were conducted. MALDI-TOF MS both markedly shortens the time to microbial species identification and concurrently expands the number of species that can be readily identified in most clinical microbiology laboratories [11, 12]. The current study is the first to assess the clinical significance of blood cultures analyzed using MALDI-TOF technology, with a species breakdown analysis of CoNS. Our findings underscore the importance of improving blood culture collection techniques and the need for accurate clinical correlation to distinguish between true infections and contaminants.

In the study period, we collected 5637 blood culture sets, with 89% yielding no bacterial growth and 11% showing positive results for a pathogen. Among the positive cultures, 77.5% were confirmed as true-positives, with 22.5% identified as false-positives. In a similar study by Weinstein et al in 1983 [5, 6] assessing hospital-wide blood cultures, the rate of contamination was 22.5% among the total positive blood culture sets and 2.3% of the total blood culture sets collected during their study period. This suggests that despite 4 decades of advances in the field of medicine, the rate of contamination remains high in the ED setting. This underscores the importance of accurate interpretation and clinical correlation when evaluating blood culture results in the ED setting as well as the need to continue focusing on improving blood culture collection techniques to prevent contamination [8, 13].

We found that the microorganisms most commonly isolated in our episodes of true bacteremia were *E coli,* followed by *S aureus*, *K pneumoniae,* and *Enterococcus* spp. This is similar to prior reports by Pien et al [4] and Weinstein et al [5], in which *S aureus* and *E coli* were the 2 most common organisms causing true bacteremia. We also observed that 30.3% of the total isolates of *S aureus* were oxacillin resistant, consistent with findings from Pien et al [4] and Lark et al [14].

As the current study included only cultures collected in the ED, the relative sources of bacteremia differed slightly from those in prior reports [4, 5]. The most frequent identifiable sources of true bacteremia were genitourinary, followed by gastrointestinal and skin. In contrast, Weinstein et al [5] noted the pulmonary tract as the most common source of bacteremia in 1983, while Pien et al [4] and Lark et al [14] reported intravenous catheters as the most frequently identified source of nosocomial bacteremia.

Previous studies [4, 5] did not analyze CoNS by species. Blood cultures positive for CoNS can be particularly challenging for providers to distinguish between as causing a true bloodstream infection or contamination. Many CoNS colonize the skin, making them common contaminants [15]. However, specific members of this variable group are now known to be nosocomial pathogens [15]. However, specific clinical and laboratory criteria can help differentiate true infections from contaminants. For instance, Elzi et al [16] found that the presence of systemic inflammatory response syndrome criteria, such as fever, tachycardia, tachypnea, and leukocytosis, significantly increased the likelihood of true CoNS bacteremia. Papadimitriou-Olivgeri et al [17] also highlighted the importance of the number of positive blood cultures and the presence of biofilm-forming genes in distinguishing true CoNS bacteremia from contamination. Some CoNS species commonly implicated in bacteremia and endocarditis include *S epidermidis, S lugdunensis, S. haemolyticus,* and *S hominis* [15, 18–20].

In the current study, *S epidermidis* was identified in 111 positive blood cultures, of which only 10 were confirmed as true bloodstream infections. Similarly, most other species of CoNS were deemed contaminants, except for *S cohnii* subsp *urealyticus*, found in 2 positive blood cultures, both confirmed as true bloodstream infections. Among CoNS, *S auricularis, S caprae, S lentus, S pseudintermedius, S saccharolyticus,* and *S warneri* were all determined to be contaminants in 100% of cases. However, the decision of whether a positive blood culture is due to contamination or a true pathogen must also be based on the clinical picture, not solely on the pathogen identified.

The patient characteristics observed in the current study align with current evidence on CoNS bacteremia. A predominance of male patients and high rates of comorbid conditions, such as diabetes mellitus and coronary artery disease, are consistent with established risk profiles for clinically significant CoNS infections [17]. The frequent presence of foreign bodies and dialysis among affected individuals supports their role as key predisposing factors, as highlighted in the literature [21]. Although CoNS are often dismissed as contaminants, studies emphasize the importance of clinical context in distinguishing true bacteremia, particularly in patients with multiple comorbid conditions or indwelling medical devices [16]. These findings reinforce the need for careful clinical assessment when interpreting positive CoNS blood cultures.

Contemporary studies have demonstrated the efficacy of ISDDs in reducing blood culture contamination. Rupp et al [22] reported a significant reduction in contamination rates from 1.78% to 0.22% with the use of specimen diversion devices in an ED setting. Similarly, Skoglund et al [23] found that routine implementation of ISDDs resulted in a cost savings of $272 per blood culture due to reduced contamination rates. Wiener-Well et al [24] observed a 31% reduction in contamination rates with the use of a diversion tube.

Our findings align with those of these studies, highlighting the importance of accurate blood culture collection techniques. The reduction in contamination rates observed with diversion devices can be attributed to the diversion of the initial blood specimen, which is most likely to contain skin contaminants. This technique does not compromise the sensitivity of detecting true bacteremia, as evidenced by the consistent rates of true-positives across studies.

The slight differences in contamination rates between our study and others may be due to variations in study design, patient populations, and implementation protocols. For instance, Nielsen et al [25] reported a 90% reduction in contamination rates with the use of Steripath, an ISDD, in an academic medical center. These variations underscore the need for standardized protocols and further research to optimize the use of ISDDs in different clinical settings.

As noted, the current study is limited by its focus on blood cultures obtained in the ED and by not including data from outpatient and inpatient settings as well as pregnant patients. This may skew the results by not capturing the full spectrum of bloodstream infections occurring in different clinical contexts, potentially limiting the generalizability of the findings compared with prior studies. A second limitation is the lack of an absolute standard for true bacteremia, as evidenced by the inability to categorize a number of positive samples as either true bacteremia or skin contamination.

In summary, our study not only analyzed contaminants by breaking them down into species but it also provides a comprehensive overview of the most common sources of true bacteremia associated with each pathogen. Hence, we believe it will shed light on making about whether to initiate antibiotics based solely on the type of the pathogen. Our findings also support the use of ISDDs to reduce blood culture contamination in the ED. These devices offer a cost-effective and clinically beneficial strategy to improve diagnostic accuracy and patient outcomes. We anticipate that this approach could help combat the problem of increasing antibiotic resistance.
