# Correction to: Retrospective Analysis of the Clinical Significance of Positive Blood Cultures in the Emergency Department: A Single-Center Study

**DOI:** 10.1093/ofid/ofag048

**Published:** 2026-02-14

This is a correction to: Farha Ahmed Karlath, Mehboob Ahmed Rehan, Annie Geiger, Michael J Mitchell, Sami Arnaout, Thomas C Greenough, Richard T Ellison, Retrospective Analysis of the Clinical Significance of Positive Blood Cultures in the Emergency Department: A Single-Center Study, *Open Forum Infectious Diseases*, Volume 12, Issue 7, July 2025, ofaf352, https://doi.org/10.1093/ofid/ofaf352

The last name of the third co-author has been corrected to read: “Annie Geiger”. The publisher apologises for this error.

10 April 2026 The following **Addendum** has been made to the correction notice above.

We erroneously originally published the correction to be of the author's “first name”, and the name was also errored to read “Anne Geiger” in the notice. “This is an addendum to the above notice 19 March 2026” has been removed and the correction notice now reads that it is the “last name” which was emended in the article to read “Geiger”. The first name of the author in the correction notice also has been emended to read: “Annie”.

